# The use of artificial intelligence to improve mycotoxin management: a review

**DOI:** 10.1007/s12550-025-00602-4

**Published:** 2025-08-08

**Authors:** M. Focker, C. Liu, X. Wang, H. J. van der Fels-Klerx

**Affiliations:** Wageningen Food Safety Research, Akkermaalsbos 2, 6708WB Wageningen, the Netherlands

**Keywords:** Machine learning, Food safety, Contaminant, Predictive modelling, Chemical analysis, Detection

## Abstract

The management of mycotoxin contamination in the supply chain is continuously evolving in response to growing knowledge about mycotoxins, shifting factors that influence mycotoxin occurrence, and ongoing technological developments. One of the technological developments is the potential for using artificial intelligence (AI) in mycotoxin management. AI can be used in various fields of mycotoxin management, including for predictive modelling of mycotoxins and for analytical detection and analyses. This review aimed to investigate the state-of-the-art of the use of AI for mycotoxin management. This review focuses on (1) predictive models for the presence of mycotoxins in commodities at both pre-harvest and post-harvest levels and (2) the detection of mycotoxins in samples by processing large datasets resulting from imaging data or chemical analyses of the sample. A systematic review was conducted, resulting in a total of 70 relevant references, including 15 references focusing on mycotoxin prediction models and 54 references focusing on mycotoxin detection, ranging from imaging to chemical analysis, and including relevant reviews. The AI applications and the most popular AI algorithms are presented. As shown by this review, AI is able to improve mycotoxin prediction models both at pre- and post-harvest levels and makes the emergence of non-invasive and fast detection methods such as imaging detection or electronic noses possible. A major challenge remains in the applicability and scalability of AI models to practical settings.

## Introduction

Mycotoxins are secondary metabolites produced by filamentous fungi, which can lead to severe health consequences for both humans and animals (Medina et al. 2017). Mycotoxin-producing fungal species can enter the food chain, pre-harvest by infecting plants or post-harvest by infecting mid or finished products, potentially producing mycotoxins (El-Sayed et al. 2022). Mycotoxin contamination is a widespread global issue, with 60% to 80% of food crops testing positive for these toxins. An estimated 25% of food crops contain at least one mycotoxin at concentrations above EU or CODEX safety limits (Eskola et al. 2020). Climate change is aggravating this problem by increasing both the frequency and severity of mycotoxin contamination in crops (Casu et al. 2024; Zingales et al. 2022).

Effective mycotoxin management requires the implementation of preventive measures and mitigation strategies throughout the entire food production chain, such as a suitable land preparation, spraying fungicides, storing crops in cool and dry places (Nada et al. 2022), processing (Suman 2021), and distributing the products in proper ways (Pavicich et al. 2025). At the pre-harvest stage, mycotoxin production is largely influenced by weather conditions in the field (Kos et al. 2023). Agronomic practices by farmers seem to also have some effect on the presence of mycotoxins in crops (Drakopoulos et al. 2021; Focker et al. 2021). At this stage, predicting the presence of mycotoxins at harvest, using data related to weather and farm agronomic practices, can further aid in mitigation efforts. Post-harvest mycotoxin contamination is influenced by mechanical injury, insect activity, and storage and processing conditions of the harvested commodities (Neme and Mohammed 2017). To minimize high levels of mycotoxins, good storage and manufacturing practices should be followed, along with Hazard Analysis Critical Control Points (HACCP) procedures. However, in case of deviations, predicting the presence of mycotoxins at the end of the storage period using data related to storage conditions can also support decision-making in mycotoxin management.

Monitoring the presence of mycotoxins in food and feed products, and their commodities, is crucial for protecting human and animals’ health from exposure to mycotoxins. This monitoring process is part of the HACCP verification and validation steps, private certification systems, and official regulatory controls. It includes collecting crop or food product samples and analyzing them for mycotoxin presence.

In recent years, artificial intelligence (AI) has been used as a novel technique in mycotoxin management, such as in predictive modelling of the presence of mycotoxins, and for detection and analyses of mycotoxins. By integrating various datasets, AI can bridge data gaps, process large datasets, and continuously improve predictions as new information becomes available (Mu et al 2024). Several reviews described the use of AI in the field of mycotoxin before. Aggarwal et al. (2024), Inglis et al. (2024), and Rayhana et al. (2025) focused on the use of AI for the detection of mycotoxins. In this review, we add the stages before detection: the prediction of mycotoxins both pre- and post-harvest.

This study aims to investigate the state-of-the-art of AI in mycotoxin management, including the prediction of mycotoxins at the pre-harvest and post-harvest stages and mycotoxin detection, summarizing the algorithms used and the accuracy of these models. A second aim is to discuss the potential future developments of AI in mycotoxin management.

## Materials and methods

A systematic literature search was conducted to explore the application of AI in mycotoxin management. Keywords were identified prior to the search on the topics of AI, mycotoxins, and prediction. The following search string was used to retrieve relevant references from the Scopus database: (“artificial intelligence” OR “machine learning” OR “deep learning” OR AI) AND (aflatoxin* OR fumonisin* OR deoxynivalenol OR ochratoxin* OR zearalenon* OR HT-2 OR T-2) AND (predict* OR forecast* OR detect*). Only studies published in the English language in the period 2000 to March 2025 were included. The citation manager Endnote was used to collect and organize the references found. The hits that resulted from the initial search were further screened by reading the title and abstract and were classified in either the group “relevant” or the group “not relevant”. The following exclusion criteria were used: (1) publication was not written in English; (2) the study was not about the prediction of mycotoxins in food commodities, for example, a paper predicting the presence of mycotoxin-producing fungi and not mycotoxins was excluded; (3) the study was not about mycotoxin management in the supply chain; and (4) studies that mentioned AI as a tool without implementing it (for mycotoxin prediction or detection purposes). After this initial classification, the material and method sections of the relevant studies were read. By reading these sections, in case a reference fulfilled one or more of the exclusion criteria, this reference, initially placed in the group “relevant”, was moved to the group “not relevant”. Snowballing was applied when reading full-text articles.

The remaining relevant references were grouped into two main topics: (a) predictive modelling, both at pre-harvest and at post-harvest level, and (b) the detection of mycotoxins. Furthermore, the following information was recorded: the year of publication, the algorithms used, the input variables used, the output of the model (a quantitative mycotoxin concentration or classes), the performance indicators used with the actual performance of the model, and the way of validation. Validation of the model was either internal, in case one dataset was split into a training, testing, and possibly a validation dataset, or external, in case the model was tested on a separate dataset, collected at a different point in time or with different conditions.

## Results

### Literature review

**Fig. 1:**
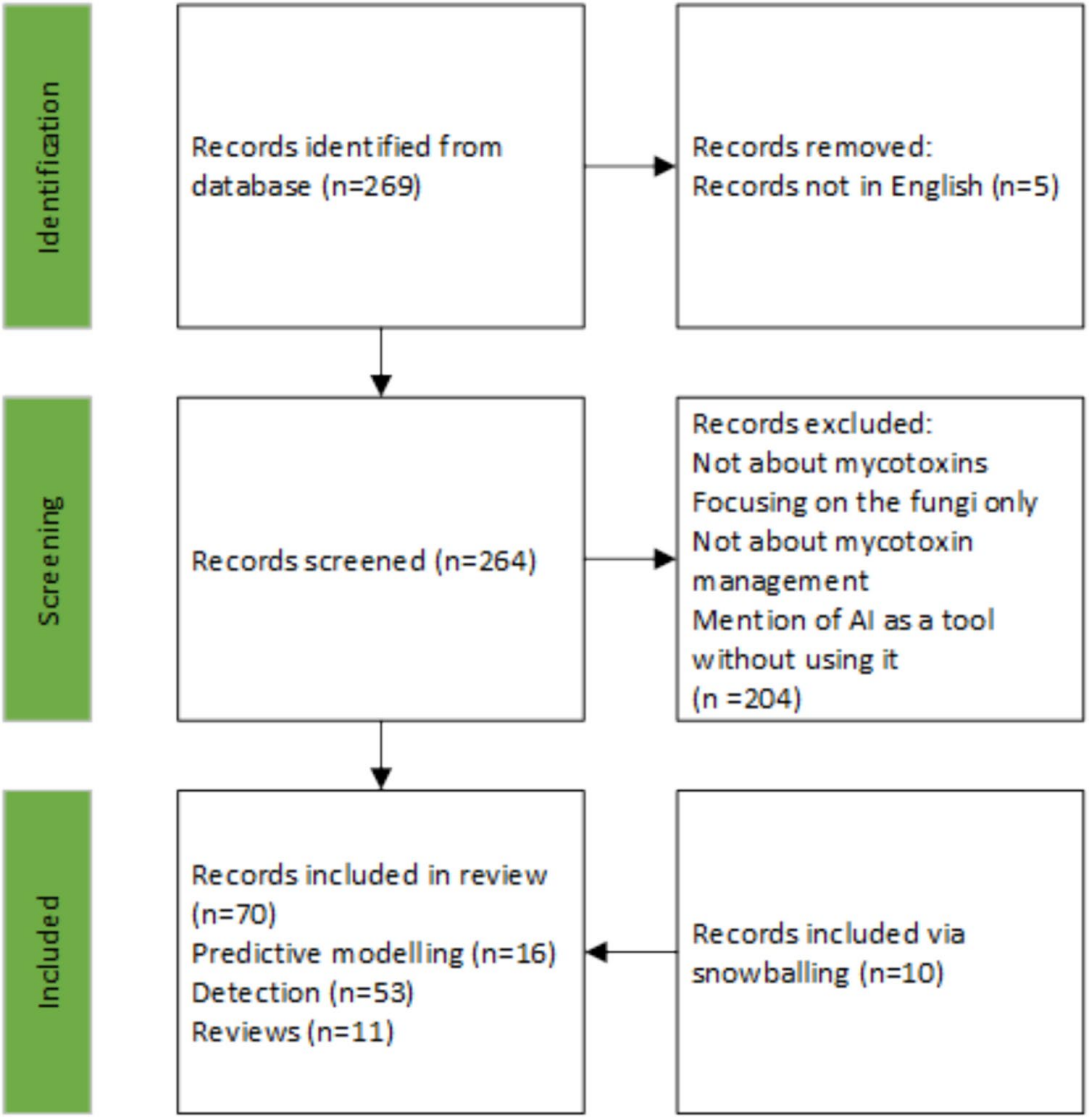
PRISMA flow diagram showing the selection process for this review

**Fig. 2:**
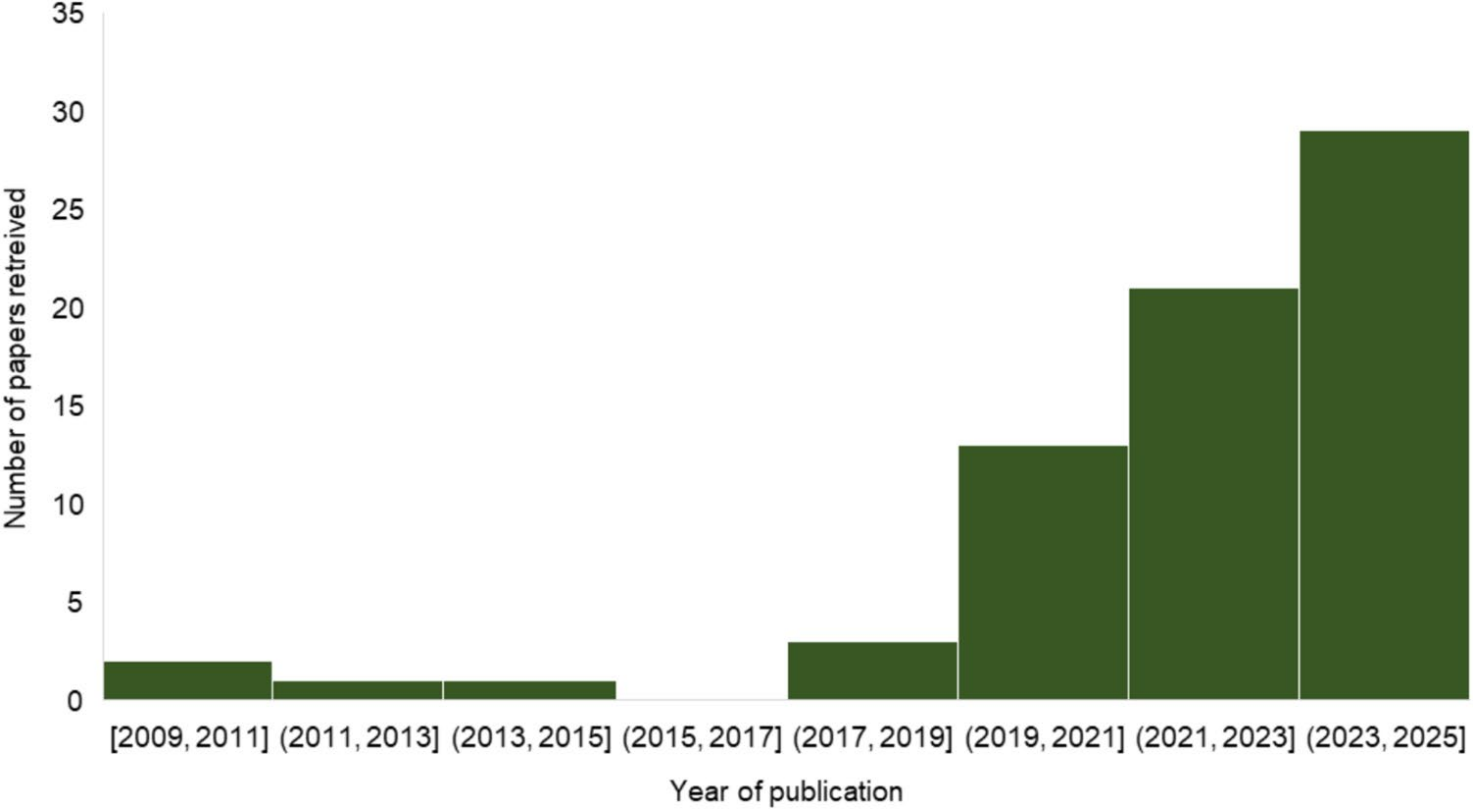
Number of papers retrieved during the time period 2009–2025 (*n* = 70)

The systematic literature search initially yielded a total of 269 references. After reading the title and abstract, 88 papers were classified as relevant. After reading the material and methods section, 60 papers remained relevant. An additional 4 original research references and 6 reviews were added via snowballing from the references of the 60 relevant papers, resulting in a total of 70 relevant papers. The relevant papers include 11 reviews, 9 papers about predictive modelling at pre-harvest level, 7 papers about predictive modelling at post-harvest and in vitro level, and 53 papers about mycotoxin detection. Figure 1 shows the selection process using a PRISMA diagram. Figure 2 shows that there has been a sharp increase in the number of papers published in the last 8 years (from 2018 onwards).

In all 59 original research papers, one or more AI algorithms have been applied (Table 1). From Table 1, it can be seen that the algorithms neural network, in different forms, support vector machine, and random forest are the most applied AI algorithms in mycotoxin management. These algorithms are used for both mycotoxin prediction and mycotoxin detection.

**Table 1:** Number of times an AI algorithm is used in the papers retrieved by the systematic search

	Prediction modelling	Detection	Total
Neural network	11	24	35
Support vector machine	2	16	18
Random forest	5	11	16
Gradient boosting	6	2	8
Partial least squares	0	6	6
Decision tree	2	1	3
Bayesian network	2	0	2
Other algorithms	1	9	10

Prediction modelling includes mycotoxin prediction pre-harvest, post-harvest, and in vitro. Detection includes all detection methods, including imaging techniques and non-imaging techniques

Table 2 shows a total of 20 regression models, with 6 regression models for in vitro mycotoxin prediction and 14 regression models for the detection of mycotoxins. The other 40 models predicted mycotoxin levels in 2 classes, so below or above a threshold (30 models), 3 classes (4 models), 4 classes (2 models), or 5 classes (5 models). Table 3 shows that models are mostly internally validated (*n* = 53), meaning in this case that a dataset collected at one point in time with similar conditions was split into training, testing, and possibly validation sets. Only 6 models were externally validated with a new dataset collected during a different year with different conditions. For regression models, the performance indicators most used were *R*^2^ (*n* = 18) and MSE (*n* = 16) (Table 3). For models predicting classes of mycotoxin levels, accuracy was almost always used as one performance indicator. Furthermore, sensitivity (*n* = 23), specificity (*n* = 17), precision (*n* = 10), and F1-score (*n* = 10) were also often reported (Table 3). The next sections describe the findings in more detail for each model purpose: prediction or detection.

**Table 2:** Type of model (regression or logistic)

	Prediction modelling	Detection	Total
Regression	6	14	20
2 classes	7	23	30
3 classes	2	2	4
4 classes	0	2	2
5 classes	0	4	4

**Table 3:** Ways of validating and expressing the performance of the models

	Prediction modelling	Detection	Total
Validation			
Internal	11	42	53
External	5	1	6
Performance indicators			
Accuracy	10	28	38
Precision	2	8	10
Recall/sensitivity	5	18	23
Specificity	5	12	17
F1-score	1	9	10
AUC	2	3	5
*R*^2^	6	12	18
(R)MSE	6	10	16
RPD	0	8	8

### Predictive modelling

AI is a relatively novel technique used in the predictive modelling of mycotoxins at harvest in the last years. As can be seen in Fig. 2, the first study found using AI was published in 2009 and the number of studies increased in the last years. Comparative analyses of using AI and with the prediction of mycotoxins using a mechanistic model—applied to the case of deoxynivalenol (DON) in wheat or aflatoxins and fumonisins in maize—showed that the application of AI improved the model’s predictive performance, with the AI model being able to predict 90% (Liu et al. 2018) or 79% (Camardo Leggieri et al. 2021a, b) of the samples correctly versus 84% (Liu et al. 2018) and 53% (Camardo Leggieri et al. 2021a, b) for the mechanistic models.

Input data used for the prediction of mycotoxins were mostly tabular data such as temperature (Wang et al. 2022a, b), water activity (aw) (Mateo et al. 2011), type and concentration of fungicides (Tarazona et al. 2021a, b), longitude and latitude (Liu et al. 2021), CO_2_ concentration (Kim et al. 2024), or soil properties (Castano-Duque et al. 2025). Examples of the use of AI in mycotoxin predictive modelling can be found both at pre-harvest and at post-harvest levels and are described in the next two sections.

#### Pre-harvest predictive modelling

A limited number of references, 8 out of 70, covered the topic of predictive mycotoxin modelling at the pre-harvest level and are described in the remainder of this section. Several studies have combined a mechanistic model with AI algorithms (Camardo Leggieri et al. 2021a; Liu et al. 2021; Castano-Duque et al. 2025 and 2023; Brandstad-States et al. 2023). Camardo Leggieri et al. (2021a) used a mechanistic model to estimate a mycotoxin risk index based on weather variables. This index was then used together with input variables related to the cropping system, such as the crop variety, the preceding crop, the sowing week, and the kernel moisture at harvest, in a deep neural network to predict the maize fields contaminated with fumonisins and/or aflatoxins above the EU legal limits, achieving an accuracy of up to 70%. In several other studies, mechanistic models were combined with Bayesian network, modelling probabilistic dependencies among variables to predict the presence of mycotoxins. Liu et al. (2021) combined a mechanistic model for aflatoxins and fumonisins to derive an aflatoxin or fumonisin risk index, which was then used as input in the Bayesian network, together with other inputs including longitudes, latitudes, and weather variables, achieving an accuracy of up to 80%. A similar approach was applied by Castano-Duque et al. (2025; 2023) achieving an accuracy of 85% and Brandstad-Spates et al. (2023) who used the aflatoxin or fumonisin risk index calculated with a mechanistic model as an input variable to their gradient boosting, neural network, or Bayesian network to estimate the mycotoxin contamination class of corn in Illinois, Texas, or Iowa, achieving an accuracy of 97%.

Other studies present a model only based on data and AI, without a combination with a mechanistic model (Liu et al. 2018; Marzec-Schmidt et al. 2021; Wang et al. 2022a, b; Sebti et al. 2025). To the best of our knowledge, the first application of a predictive model for mycotoxins that was only based on AI (not combined with a mechanistic model) is a Bayesian network to predict the presence of DON in wheat grown in the Netherlands. This model achieved an accuracy of 90% (Liu et al. 2018). Authors used a dataset of DON concentrations in Dutch wheat fields collected for more than 10 years. Weather data and agronomic variables such as the use of fungicides were used in the Bayesian network to predict the class (low/medium/high) of DON contamination. Marzec-Schmidt et al. (2021) modelled the effect of weather conditions to classify the contamination of grains (barley, wheat) with DON in several countries in the Baltic Sea region, using and comparing the four AI algorithms of decision tree, random forest, support vector machine. Inputs used in these algorithms were mainly weather variables such as precipitation, relative humidity, temperatures, wind speed, and wind direction. The accuracy of the model was 77%. Wang et al. (2022a, b) predicted the probability of mycotoxin contamination of wheat in Europe at the regional level, using a random forest algorithm, achieving an accuracy of up to 99%. In addition to monitoring data on various mycotoxins in wheat, input data for this model included wheat phenology data, weather data, and satellite spectral data per grid in Europe. Sebti et al. (2025) explored a neural network to classify the potential for ochratoxin contamination of grapes grown in Italy using mainly weather data as input. The neural network model was trained and validated using generated datasets (not from real fields) of five classes, representing the probability of ochratoxin A production. The model achieved an overall accuracy of 99%.

#### *Post-harvest and *in vitro* predictive modelling*

A total of 7 out of 70 papers focused on mycotoxin prediction at post-harvest level and in vitro data modelling (Kim et al. 2024; Mateo et al. 2009, 2011, 2021; Srinivasan et al. 2022; Tarazona et al. 2021a, 2021b). At post-harvest level, the studies presenting prediction models for mycotoxins based on AI are scarce (one reference) and apply to storage only. Kim et al. (2024) used a neural network, based on CO_2_ respiration rate and the visual appearance of mould formation in an experimental stored wheat batch, to predict if stored wheat was contaminated with DON or aflatoxins, and achieved a model accuracy of 83%.

Several studies investigated the prediction of fungal growth and the production of mycotoxins in a laboratory setting using AI to analyze the data. Srinivasan et al. (2022) used the algorithms neural network, regression tree, and random forest to model the growth of *Fusarium culmorum* and the production of zearalenone and fumonisins based on aw and temperature in milled maize kernels. The highest achieved *R*^2^ was 0.95. Similarly, Tarazona et al. (2021a; 2021b) used the algorithms neural network, random forest, and extreme gradient boosting to predict the growth of *Fusarium* spp. and the production of zearalenone and fumonisins based on temperature and aw in milled maize kernels in vitro. These models were then used to estimate the effect of various concentrations of different fungicides on mycotoxin production in maize extract medium, with an *R*^2^ of 0.9 (Tarazona et al. 2021a). The same AI model for predicting the growth of *Fusarium* spp. and the production of zearalenone and fumonisins was used to predict the effect of essential oils that can be applied as a food preservative during storage, with an *R*^2^ of 0.9 (Tarazona et al. 2021b). A similar approach was used by Mateo et al. (2021), applying various machine learning algorithms (XGBoost, random forest, support vector machine, and neural network) to predict the growth of *Fusarium sporotrichioides* and the production of T-2 and HT-2 in oat grains in vitro under different temperatures, aw, and using different ethylene–vinyl alcohol copolymer films containing pure essential oils. An *R*^2^ of 0.9 was achieved. Similarly, a multi-layer perceptron (MLP) artificial neural network has been used to predict ochratoxin A formation in a grape juice-based medium by *Aspergillus carbonarius* (*R*^2^ of 0.99) (Mateo et al. 2009) and deoxynivalenol in barley grain contaminated with *Fusarium culmorum* (*R*^2^ of 0.96) (Mateo et al. 2011). All these studies used experimental data obtained in a laboratory setting as input for AI algorithm training.

### Mycotoxin detection

Using our search strategy, 54 out of 70 references were placed in this section, the detection of mycotoxins in (post-)harvest samples. In recent years, AI has been increasingly used for the detection of mycotoxins, with the first model published in 2012 and 17 models published in 2024 (Fig. 2). The techniques are divided into two sections, depending on the input data used for the modelling: spectral imaging (spectral data) (*n* = 46) and non-spectral detection methods (sensor data) (*n* = 8).

#### Spectral imaging

Non-invasive detection techniques such as spectral imaging are upcoming techniques with the possibility to screen mycotoxins in bulk samples on-site or in-line. AI was used to process the spectral data and perform band selection with the aim to classify the samples into high or low contaminated samples at a chosen threshold for the presence of mycotoxins. Multiple AI algorithms were used, ranging from classical machine learning algorithms to deep learning algorithms. Most studies tried multiple algorithms and selected the best performing one(s). To date, the applied imaging techniques include fluorescence imaging, the visual range: red-blue-green (RGB) imaging, infrared spectroscopy, and full hyperspectral imaging (HSI). These imaging techniques capture spectral signatures, focusing on different wavelengths, from short to long. For more information on these imaging techniques and a more complete overview of literature, we refer to the reviews of Freitag et al. (2022), Femenias et al. (2022), and Guo et al. (2025).

Fluorescence imaging has been used in combination with similar AI algorithms, including support vector machine (Bertani et al. 2020; Kalkan et al. 2014; Kılıç et al. 2024; Wang et al. 2025), random forest (Kabir et al. 2024; Wang et al. 2025), and neural networks (Bertani et al. 2024; Kalkan et al. 2014; Kılıç and İnner 2022; Liu et al. 2025; Sadimantara et al. 2024a, 2024b). These models all achieved high accuracies of over 90%.

One study showed that RGB images can be implemented in practical applications. Leo (2023) has developed a mobile application utilizing deep learning to classify mycotoxin contamination in maize kernels using mobile phone pictures. A neural network was trained on image data from stored maize in eight different storage facilities across four regions in Tanzania. The developed model was tested in practice settings, showing good performance with a reported accuracy of 100%.

Near-infrared has been used, amongst others, for the detection of aflatoxins in chili pepper and maize, zearalenone in maize and wheat, deoxynivalenol in wheat, and enniatins in wheat. Again, the top three most used algorithms to classify the samples (different classes of contamination), based on the near-infrared data, were random forest (Almoujahed et al. 2025; Ghilardelli et al. 2022; Sein et al. 2024), support vector machine (Kim et al. 2023; Sein et al. 2024; Zheng et al. 2020), and neural networks (Liu et al. 2022; Wang et al. 2022a, b; Zhao et al. 2024). Best performing algorithms achieved accuracies above 90% (Almoujahed et al. 2025; Ghilardelli et al. 2022; Kim et al. 2023; Liu et al. 2022; Wang et al. 2022a, b; Zhao et al. 2024; Zheng et al. 2020) or above 70% (Sein et al. 2024). Ji et al. (2024) used a variety of alternative AI algorithms such as kernel-based extreme learning machine and the Harris Hawks optimization algorithm to quantify zearalenone in wheat with an *R*^2^ of 0.99.

Hyperspectral imaging has been applied for classification of contamination of aflatoxins in almonds, chili pepper, peanuts, and deoxynivalenol in barley, maize, and wheat. The top three algorithms used to classify the samples based on the hyperspectral data were neural networks (Han and Gao 2019; Liu et al. 2024; Saini et al. 2024; Zhao et al. 2025; Zhu et al. 2022a, 2022b, 2024), random forest (Kabir et al. 2024; Öner et al. 2019; Saini et al. 2024; Wang et al. 2024a, b, c; Zhu et al. 2023), and support vector machine (Öner et al. 2019; Saini et al. 2024; Su et al. 2021; Wang et al. 2024a, b, c; Zhongzhi and Limiao 2020). Best performing algorithms achieved accuracies well above 90%. Ataş et al. (2012) used multi-layer perceptrons to classify samples below or above a threshold, with an accuracy of 87.5%.

A related approach is surface-enhanced Raman spectroscopy (SERS) in combination with AI that is also an emerging detection technique to directly detect, potentially on-site, the presence of mycotoxins in food and feed. Today, the application of this technique is limited to a laboratory setting (Wu et al. 2021). Convolutional neural networks are mostly used to interpret the data generated by this technique and to make a prediction regarding the level of mycotoxin contamination. The achieved *R*^2^ were all above 0.98 (Deng et al. 2022; Weng et al. 2021; Xue et al. 2024). This technique was also combined with a lateral flow system for the detection of DON, with an accuracy of 98.8% (Sun et al. 2025). These authors used a series of assay solutions containing DON standards coupled to a nanoprobe. Raman signals were used to classify different DON concentrations using an artificial neural network and the k-nearest neighbours algorithm. For more information about this technique and a complete overview of the literature, we refer to the reviews of Wu et al. (2021) and Logan et al. (2024).

#### Non-imaging detection methods

In addition to the use of spectral information to detect mycotoxins in samples, the use of an electronic (E-) nose in combination with AI can be used. An E-nose is able to capture volatile organic compounds, providing a fingerprint of the sample analyzed. This fingerprint can further be interpreted with AI. Camardo Leggieri et al. (2022) used the electronic nose for identification of wheat samples contaminated with DON. The classification tree was used to categorize samples according to four DON contamination thresholds, with an accuracy of 83%. Another study combined E-nose and an artificial neural network (ANN) to assess the contamination of aflatoxin B_1_ and fumonisins (above or below the legal limit) in maize, with an accuracy of almost 70% (Camardo Leggieri et al. 2021b). Wang et al. (2024a, b, c) used an electronic nose equipped with 10 metal oxide sensors to predict if petfood samples had zearalenone levels above the Chinese legal limit. The algorithms linear regression, k-nearest neighbours, support vector machines, random forest, XGBoost, and multi-layer perceptron (MLP) were used to classify the samples based on their volatile profiles. This technique achieves a high accuracy for the quantification of DON (accuracy of about 98%).

Other emerging non-invasive techniques include a microwave detection technology coupled with AI. This technique has been applied to the detection of aflatoxins in wheat. The algorithms supporting machine vector, random forest, or convolutional neural network have been used for data analyses (Deng et al. 2023; Xu et al. 2022). Xu et al. (2022) prepared samples of dried wheat grains with different degrees of mildew. The microwave detection device compares the amplitude information and phase information of the sampled signal and the reference signal and expresses it by a voltage value. Several multivariable analysis algorithms were used, including support vector machine, extreme learning machine, random forest, and partial least squares, to predict the mycotoxin contamination class. An *R*^2^ of 0.97 was achieved. Deng et al. (2023) used a similar technique and prepared in the same way a multitude of mouldy wheat samples. A neural network model was used to classify the aflatoxin concentration of the samples, with an accuracy of 100%.

Biosensors, recognizing elements such as enzymes, antibodies, or DNA fragments, are members of another type of emerging rapid detection method. Biosensors can be electrochemical, optical, thermal, or mass sensitive. Pisoschi et al. (2024) described the use of biosensors to detect mycotoxins. This technique was used in combination with AI algorithms, and, in particular, the random forest algorithm, to interpret the data (Ma et al. 2023).

## Discussion

This review shows the increasing trend of research related to using AI for mycotoxin management, for both pre- and post-harvest predictive modelling and analytical detection of mycotoxins (Fig. 2). AI-based models were developed for mycotoxin management by enabling early forecasting of mycotoxin presence, both at pre- and post-harvest levels, and enhancing the efficiency of mycotoxin detection in samples.

A multitude of AI algorithms were used, with neural networks, support vector machine, random forest, and gradient boosting as the most popular algorithms (Table 1). In the field of predictive modelling, these AI algorithms are capable of capturing complex, non-linear relationships among multiple environmental, agronomic, and biological variables that may influence mycotoxin contamination. In this way, the potential influence of many different variables and their underlying relationships can explain the phenomenon. In the field of mycotoxin detection, the same also holds: AI algorithms are able to deal with the high number of parameters and data from spectral and non-spectral imaging methods for analytical detection.

This review also shows that the currently used AI algorithms for mycotoxin management are mostly using tabular data, image data, or sensor-based data. Satellite imagery and remote sensing technologies can provide large-scale monitoring data of environmental conditions and land use, enabling AI to predict high-risk areas and/or conditions for mycotoxin contamination. At the pre-harvest level, the addition of satellite images in mycotoxin prediction models can be used to extract the crop growing areas and to identify crop phenology stages, in order to improve the performance of the predictive models. For example, satellite images were used to generate a crop mask layer for maize acreage (identifying where maize is grown) each year to develop geospatial modelling of mycotoxin contamination (Castano-Duque et al. 2023). Wang et al. (2022a, b) used satellite image data from the period of wheat flowering to wheat harvest to derive vegetation indexes as extra predictors in their prediction model for the presence of DON in wheat at harvest based on AI. AI segmentation algorithms, such as unsupervised clustering and deep learning–based image segmentation, can filter out irrelevant background elements (e.g. soil, leaves) and concentrate analysis on biologically meaningful structures—such as wheat ears, which are most susceptible to Fusarium head blight (FHB) and consequent mycotoxin accumulation (Wang et al. 2024a, b, c). Data from other sources, such as text data, have not yet been used for mycotoxin management. Text data combined with AI algorithms has been used in other domains. For instance, natural language processing (NLP) was applied to extract insights from scientific literature and regulatory reports, facilitating cross-referencing of analytical results with evolving food safety standards (Song et al. 2022). Such a use of text-based data with the relevant AI algorithm could be an advancement for mycotoxin management as well. Multimodal AI, machine learning models capable of processing and integrating information from multiple types of data (such as tabular, image, and text data), could be applied to improve model accuracy and reliability (Yu et al. 2023).

Our results show that all references report an excellent model performance, especially when accuracy, mostly above 90% or *R*^2^, mostly above 0.9, is considered. However, models tend to score much lower when other performance indicators measuring how good the model is at avoiding false negatives, such as the recall or sensitivity, are considered, an important aspect when considering the prediction or detection of mycotoxin levels. The model of Marzec-Schmidt et al. (2021) shows, for example, an overall accuracy of 77% for the prediction of DON in Swedish spring barley with a random forest model but a sensitivity of 63%. This shows that accuracy does not always give the full picture of the performance of the model. Not all models retrieved in this review consider another performance indicator than the overall accuracy.

Our results show that only six models were externally validated with a dataset collected at a different time point with other conditions (Table 2) leading to major challenges in model applicability and scalability. Most AI models have been developed in research studies but have not yet been applied under real-life conditions. Their added value in practice has yet to be demonstrated. It is essential to validate the developed AI models in operational environments that account for real-world variability, including, for instance, sample noise, seasonal changes (e.g. the weather shows different patterns each year), regional differences, and cultivar-specific traits. For example, imaging techniques, up to now mostly developed for post-harvest mycotoxin prediction in laboratory settings, should in practice be usable in storage facilities. On-site storage conditions will introduce additional sources of variability to the sample, such as background, movements, or impurities that the AI-driven models need to be able to handle. The biggest challenge to increase model scalability is the limited availability of high-quality data for developing AI-driven models.

Addressing this limitation of model applicability in real life will require a multifaceted approach, including improving the collection and access to high-quality and diverse datasets, promoting adherence to FAIR principles (Findable, Accessible, Interoperable, and Reusable) (Inau et al. 2021), developing standardized data collection and annotation protocols, and implementing methods such as federated learning (Zhang et al. 2021) to enable secure and privacy-preserving collaboration across institutions. These aspects will benefit from the involvement of relevant stakeholders throughout the entire process, from data collection to AI model development. This requires strengthening interdisciplinary collaborations between AI researchers, food scientists, and risk managers from private and public organizations to ensure that AI-driven models are based on the best quality data possible and that models align with global food safety standards and mycotoxin management needs. Furthermore, the integration of explainable AI (XAI) techniques would enhance model transparency and stakeholder trust. XAI provides visuals, a textual explanation, or some examples to improve the understanding of the models (van der Velden 2024; Wang et al. 2024a, b, c).

In our opinion, mycotoxin forecasting models at the pre-harvest level have the potential to be used in practice. Several of these models have been externally validated. These models can be integrated into dashboard systems that buyers or food companies use to either make choices on sourcing or on their (risk-based) monitoring plans. One example is the DSM mycotoxin prediction tool (https://mycotoxinprediction.net/login/). The models used for these predictions should continuously be updated and validated when new data becomes available. AI models focusing on the detection of mycotoxins need to be externally validated in practical settings, with different varieties of crops and different measuring times, before having the potential to be used in practice. External validation with independent datasets is needed, and performance indicators should be carefully selected and fully reported to ensure the quality of the prediction model. Furthermore, when extracting information from the references, it was often difficult to extract the output considered, such as the mycotoxin concentration or classes of mycotoxin levels. Many references focused on the algorithms and their performance, indicating that the practical application is not yet considered. 

To conclude, as shown by the current review, during the last 8 years, AI has been increasingly used and shown its potential to improve mycotoxin management. AI can improve mycotoxin prediction models both at pre- and post-harvest levels by handling large datasets and integrating multiple types of input data. AI can also analyze the large datasets produced by emerging non-invasive detection methods for mycotoxins such as imaging techniques or electronic noses. A major challenge remains in the applicability and scalability of AI models to practical settings. Mycotoxin prediction models are not widely used in practice yet. Detection methods integrating the use of AI are developed in laboratory settings and need to be tested in a practical setting.

## Data Availability

No datasets were generated or analysed during the current study.
